# Deep Learning Techniques for Prostate Cancer Analysis and Detection: Survey of the State of the Art

**DOI:** 10.3390/jimaging11080254

**Published:** 2025-07-28

**Authors:** Olushola Olawuyi, Serestina Viriri

**Affiliations:** School of Mathematics, Statistics and Computer Science, University of KwaZulu-Natal, Durban 3629, South Africa; 221119659@stu.ukzn.ac.za

**Keywords:** prostate cancer, prostate cancer detection, deep learning, survey of state of the art

## Abstract

The human interpretation of medical images, especially for the detection of cancer in the prostate, has traditionally been a time-consuming and challenging process. Manual examination for the detection of prostate cancer is not only time-consuming but also prone to errors, carrying the risk of an excess biopsy due to the inherent limitations of human visual interpretation. With the technical advancements and rapid growth of computer resources, machine learning (ML) and deep learning (DL) models have been experimentally used for medical image analysis, particularly in lesion detection. However, several state-of-the-art models have shown promising results. There are still challenges when analysing prostate lesion images due to the distinctive and complex nature of medical images. This study offers an elaborate review of the techniques that are used to diagnose prostate cancer using medical images. The goal is to provide a comprehensive and valuable resource that helps researchers develop accurate and autonomous models for effectively detecting prostate cancer. This paper is structured as follows: First, we outline the issues with prostate lesion detection. We then review the methods for analysing prostate lesion images and classification approaches. We then examine convolutional neural network (CNN) architectures and explore their applications in deep learning (DL) for image-based prostate cancer diagnosis. Finally, we provide an overview of prostate cancer datasets and evaluation metrics in deep learning. In conclusion, this review analyses key findings, highlights the challenges in prostate lesion detection, and evaluates the effectiveness and limitations of current deep learning techniques.

## 1. Introduction

Prostate cancer ranks among the leading causes of death in men worldwide. This has been confirmed by several international cancer organisations, which recognise it as among the most prevalent cancers affecting men globally [1]. The World Cancer Report 2014 by Stewart and Wild remains a landmark publication which provides an extensive and authoritative overview of the global cancer challenge, its causes, and prevention and control techniques [1]. In the United States, prostate cancer is the second leading cause of cancer-related death in men, following lung cancer. Approximately 1 in 44 men die of prostate cancer, with African American men and those of Caribbean descent being at a significantly higher risk of developing the disease compared to men of other racial groups [2]. The American Cancer Society projects approximately 299,010 new cases of prostate cancer and 35,250 related deaths in 2024 [2]. Recent advances in machine learning and computer vision have significantly enhanced computer-aided diagnosis (CAD) and early detection systems for prostate cancer [3,4].

The detection of prostate cancer has traditionally relied on manual screening and visual inspection. However, these methods involve pathologists visually examining lesion images and are often time-consuming, complex, and susceptible to errors. This is mainly due to the complexity of prostate lesion images [5,6,7]. The clear identification of lesions is essential for accurately analysing, interpreting, and understanding lesion images. Nevertheless, this process can be challenging due to several factors such as data scarcity, lesion variability, model generalisation, and the need for standardised, interpretable, and clinically applicable solutions [8,9]. The images of prostate lesions frequently pose a challenge for accurate recognition. The presence of blood vessels and other noise can interfere with the detection of the lesion. Additionally, the low contrast in specific medical images of the prostate lesion zone can increase the difficulty of obtaining accurate detection. In addition, prostate lesions have a wide range of diameters, morphologies, and colours, which limits the potency of some diagnostic approaches when aiming for high accuracy. In imaging techniques such as multiparametric MRI (mpMRI) or ultrasound, variations in lesion properties can compromise sensitivity and specificity, making it challenging to distinguish between benign and malignant lesions [10].

In prostate imaging modalities such as mpMRI and ultrasound, lesion heterogeneity encompassing variations in size, shape, anatomical location, and internal composition frequently produces overlapping imaging signatures between malignant and benign entities (e.g., BPH, prostatitis, fibrosis), particularly in the transitional zone, which degrades both sensitivity and specificity [11]. As a direct consequence, acquisition protocols such as the selection of b values in DWI, timing and dosage of contrast agents in DCE, and rectification of motion or metal artefacts become inconsistent across studies and imaging systems, introducing labelling uncertainty and inter-reader variability, which, in turn, undermines dataset quality [12].

These inconsistencies generate heterogeneous image quality and ambiguous annotations, two critical impediments to developing robust deep learning models: Without standardised, high-fidelity imaging and unequivocal ground truth, AI systems risk learning spurious features, failing to generalise across centres, and underperforming on rarer but clinically significant lesion types. Therefore, advancing protocol harmonisation, deploying automatic motion/artefact correction, and integrating multimodal fusion (e.g., mpMRI + ultrasound) are essential steps toward generating high-quality, standardised imaging databases that support clinical-grade AI development and deployment [13].

**Figure 1:**
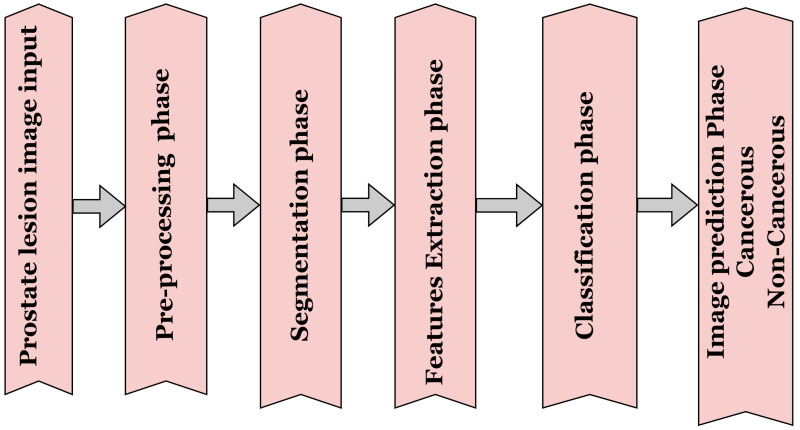
Pipeline for cancer diagnosis of prostate lesion image analysis.

Pathologists find the manual screening of prostate lesions challenging, which has led to the need for automated computerised diagnostic systems. Such systems have helped analyse prostate lesions and support pathologists in making accurate and rapid decisions [14,15]. By enabling the early and precise detection of prostate cancer, these technologies have significantly contributed to informed clinical decision-making [15]. Prostate disorders, such as prostate cancer, are highly fatal, but if detected early, they are treatable [16,17]. Over the years, computerised techniques have been created to increase the diagnosis and detection of prostate cancer. Traditional medical image analysis typically follows a sequence of low-level image processing steps. As shown in Figure 1, prostate cancer detection and diagnosis generally involve several key stages like image pre-processing, feature extraction, segmentation, and lesion classification [18,19].

The pre-processing phase involves various methods, including contrast or intensity adjustments, morphological operations, binarisation, colour or grayscale transformations, and data augmentation. At this stage, image noise and other artefacts are removed [20]. Image resizing is also applied to make images standard and reduce computational complexity. After pre-processing, image segmentation identifies areas of interest (AOI) in both affected and healthy regions [19]. In this step, normal tissues are removed to facilitate accurate feature extraction from the lesions, ensuring a reliable diagnosis [8,21].

Conventional segmentation methods, such as clustering, thresholding, and edge and region-based approaches, have been widely used for prostate lesion analysis in the context of cancer detection [22,23]. However, these approaches also have limitations regarding the discriminative representation of the complex visual elements of prostate lesions and often perform poorly in lesion extraction [7,18].

Over the past few years, intelligent segmentation techniques have been widely implemented, including fuzzy logic, artificial neural networks (ANNs), genetic algorithms, and, more recently, deep learning methods, to produce accurate and robust segmentation of prostate lesions [24,25]. Using images to analyse the state of the prostate, techniques are employed to extract lesion-specific features. Classically used feature extraction methods are image transformation, template matching, projection histograms, graph analysis, contour-based methods, gradient features, Fourier descriptors, and Gabor filters [26].

The extracted features are then processed by classification methods to predict the presence or absence of cancer in the targeted prostate areas. Traditional prostate lesion image classification techniques include fuzzy classification, decision trees, artificial neural networks (ANNs), and support vector machines (SVMs) [22,27].

Deep learning methods have recently achieved state-of-the-art performance in prostate lesion analysis and can even predict prostate diseases using imaging data [12,28]. Their success lies in their ability to learn and extract deep hierarchical features from complex image datasets [29]. For example, deep convolutional neural networks (DCNNs) are a popular deep learning method known for their ability to handle complex tasks. They are especially good at spotting fine details, which plays a key role in identifying prostate lesions [30]. This advanced technique outperforms handcrafted features by effectively extracting high-quality features from entire prostate lesion images [31,32,33]. The key contributions of this survey study are as follows:

1. This survey thoroughly reviews state-of-the-art deep learning techniques applied to prostate lesion analysis and detection, offering insights into the strengths and limitations of various methods.
2. It categorises existing techniques based on their applications, such as lesion detection, segmentation, and classification, helping researchers understand the landscape of solutions.
3. The study identifies critical challenges in prostate lesion analysis, including imaging modalities, image quality, variation in acquisition protocols, patient-specific factors, data annotation, and ground truth, thereby guiding future research directions.
4. In this study, we proposed several deep learning-based models that exhibit exceptional performance in prostate lesion segmentation. Additionally, we conducted a comprehensive review of various architectures, including V-Net, fully convolutional networks (FCNs), Attention U-Net, cascaded FCNs, and deep fully convolutional residual networks (FCRNs).
5. By showcasing real-world implementations, this paper bridges the gap between research and clinical practice, demonstrating the potential impact of deep learning in improving prostate cancer diagnosis and treatment.
6. The survey discusses commonly used datasets and evaluation metrics, providing a benchmark for researchers to assess the performance of their models.

### Challenges in the Detection of Prostate Cancer

Detecting prostate cancer is challenging due to the variability in image types and sources. This variability creates significant challenges in obtaining reliable and accurate detection [34]. The following are some factors that play a role in the problems listed:

1. Imaging Modalities: Several imaging sequences, such as T1-weighted MRI, T2-weighted MRI, dynamic contrast-enhanced (DCE) MRI, and diffusion-weighted imaging (DWI), provide complementary information about prostatic tissue. Each modality targets different tissue features, thus posing difficulties in merging information from other sources to obtain accurate lesion detection. Although the sensitivity for lesion types among specific modalities may be more significant, it generally lacks specificity and can lead to differences in detectability results.
2. Image Quality: Variations in image quality, including differences in resolution, contrast, and noise, can also significantly impact the detectability of lesions. High-quality, high-contrast images can reveal finer details, while low-quality, lower-contrast images can obscure subtle irregularities. Differences in hardware, scanning parameters, or operator procedures may exacerbate variations in image quality, thereby affecting the accuracy of analysis.
3. Variation in Acquisition Protocols: MRI acquisition protocols vary between institutions, leading to image appearance and quality variability. Differences in scanner type (e.g., 1.5 T vs. 3 T magnetic field strength), slice thickness, and imaging parameters can lead to variability in the data acquired. These contradictions challenge the development of general models of lesion detection that generalise well to a range of data.
4. Patient-Specific Factors: Patient-specific variables, such as anatomy, age, prostate volume, and the presence of benign lesions (benign prostatic hyperplasia or inflammation), contribute to image variability. These can make it difficult to standardise so that benign and malignant lesions can be differentiated reliably.
5. Data Annotation and Ground Truth: Inconsistencies in image annotation and labelling between datasets can affect the training of artificial intelligence models. For example, non-standardised, discriminative, conditional, and subjective annotations by various radiologists can lead to inconsistencies in ground truth, thus affecting the performance of detection algorithms.

## 2. Methods for Analysing Prostate Lesion Images

### 2.1. Pre-Processing Methods for Prostate Lesion

The pre-processing methods include (i) contrast normalisation that extends the image histogram to make it clear, (ii) intensity normalisation, and (iii) histogram equalisation, where all three methods are used to make the image high-quality [35]. Furthermore, binarisation transforms a grayscale image into black and white, compressing the image’s information from 256 grey shades to several black-and-white formats. Morphological operations, including erosion and dilation, are further used for feature extraction and identifying object positions in the image [36,37].

### 2.2. Segmentation Method for Prostate Lesions

Image segmentation is a crucial step in the automated detection and diagnosis of prostate lesions. This is an essential pre-processing step in analysing prostate lesion images, as it separates lesions from normal tissue to select regions of interest. In this subsection, several segmentation methods for prostate lesions are reviewed, including handcrafted feature-based methods (edge detection and contour features), intensity-based methods, region-based methods, texture-based methods, and intelligence-based supervised methods [38,39,40,41,42]. Complicated intelligent strategies for segmenting prostate lesions utilise artificial neural networks (ANNs) and deep learning (DL) methods [43,44]. Table 1 summarises the strengths and weaknesses of various segmentation techniques.

### 2.3. Traditional Intelligence Method

This system utilises artificial intelligence to analyse images by learning, reasoning, and perceiving from large existing image datasets. This method uses supervised and unsupervised approaches for prostate lesion segmentation where the system is trained and learns from available datasets [51]. Standard AI-based segmentation methods include artificial neural networks (ANNs) [42], fuzzy c-means (FCM), and genetic algorithms [52,53]. Recently, DL techniques have also been integrated into these AI-based methods.

### 2.4. Deep Learning-Based Method

**Figure 2:**
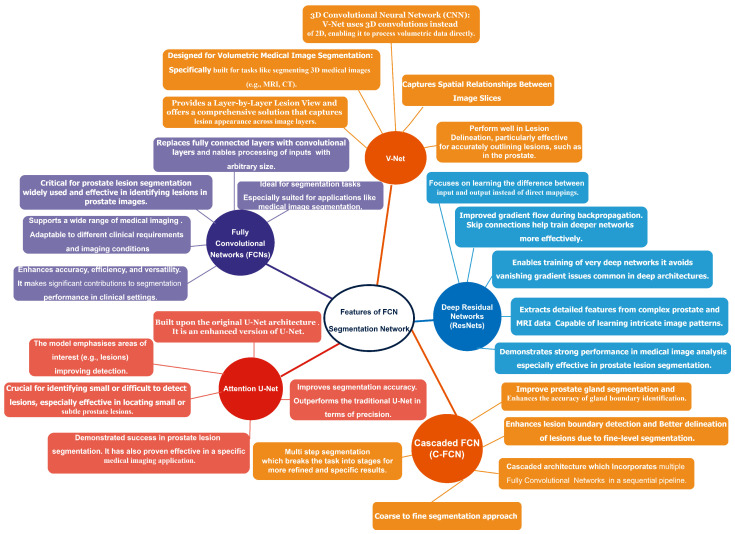
Deep learning-based models for image segmentation [54,55,56,57].

Deep learning methods have made significant advancements in segmenting prostate lesion images, a challenging task in computer vision [43]. Researchers have proposed various deep learning-based models demonstrating outstanding performance in prostate lesion segmentation [45]. Figure 2 below summarises features of some of the architectures, like V-Net, fully convolutional networks (FCNs), Attention U-Net, cascaded FCNs, and deep fully convolutional residual network (FCRNs).

## 3. Classification Techniques for Prostate Lesions

The classification of prostate lesion images plays a crucial role in prostate cancer detection. Lesion classification, particularly medical image-based classification, plays an essential role in diagnosing and evaluating prostate cancer. Over time, various conventional and machine learning-based techniques have been used to improve the accuracy and effectiveness of prostate lesion classification. This subsection describes both classical and state-of-the-art classification approaches.

### 3.1. Traditional Methods of Prostate Lesion Classification

The conventional method to classify prostate lesions mainly consists of the extraction of handcrafted features from medical images like CT, MRI, and ultrasound [58]. These features, including shape, texture, intensity, and size, can be used to classify benign and malignant tissues [59]. Methods such as simple vector machines (SVMs), k-nearest neighbors (KNN), decision trees, and fuzzy logic provide fascinating insights into the characterisation of lesions. Despite their reliance on explicit feature extraction, these methods are still valuable especially for limited or medium-sized datasets [31]. However, they often struggle with complex or noisy data and may have difficulty generalising across diverse patient populations without extensive manual intervention. A comparison of traditional techniques commonly employed for classifying prostate lesions is shown in Table 2 below.

### 3.2. Deep Neural Networks: A Cutting-Edge Technology

**Figure 3:**
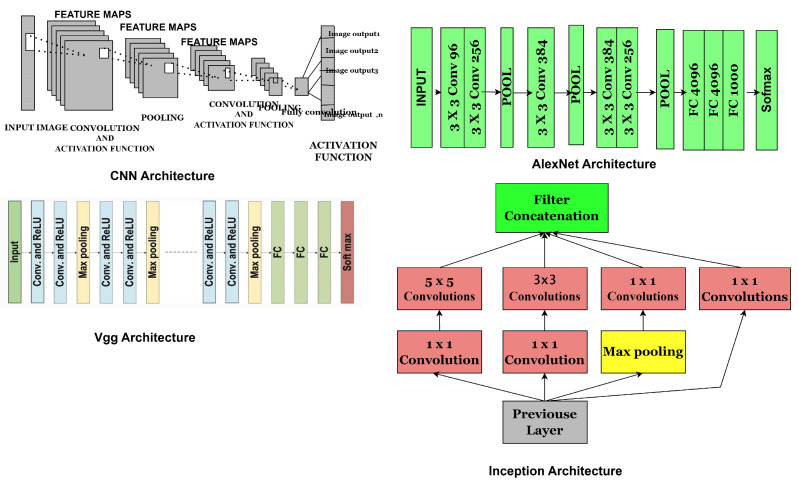
CNN architetures.

1. The exponential growth in computing power, particularly with advancements in graphical processing units (GPUs), as predicted by Moore’s Law in 1971, has driven rapid progress in computer vision technology, primarily in the development of deep learning (DL) architectures, such as convolutional neural networks (CNNs). These advancements and efficient formulations in deep learning (DL) architecture have yielded exceptional state-of-the-art performance in image processing and classification. These approaches have shown better performance than traditional techniques. However, there are still challenges in the use of deep learning-based image analysis.
2. Convolutional Neural Network Components: CNNs are artificial neural networks (ANNs) primarily designed for image analysis. These networks consist of neurons that can optimise themselves through learning and adaptation. CNNs comprise several layers, each serving a different function, and can process high-dimensional input vectors, such as images. The general architecture of CNN is illustrated in Figure 3, highlighting key components, including layers, activation functions, and hyperparameters. CNNs layers are typically categorised into convolutional, pooling, and fully connected layers. The activation function is a mechanism that transforms input signals into output signals, playing a crucial role in the functioning of neural networks. Standard activation functions include linear activation, Sigmoid functions, and rectified linear units (ReLUs), also known as piecewise linear functions, exponential linear units (ELUs), and Softmax, which are mathematically represented by the equations below.

### 3.3. Exponential Linear Unit (ELU)

The ELU function introduces a slight negative slope for inputs below zero, making it more robust to noise and allowing for a smoother gradient than other activations, such as ReLU.

(1) $$ELU\left(x\right)=\left\{\begin{array}{cc} x, & \mathrm{if}\;x\geq 0\\ \alpha (e^{x}-1), & \mathrm{if}\;x<0 \end{array}\right.$$

where α is a positive constant (typically 1) that controls the value to which ELU saturates for negative inputs.

### 3.4. SoftMax Function

(2) $$\sigma \left(z_{i}\right)=\frac{e^{z_{i}}}{\sum_{j=1}^{N}e^{z_{j}}}$$

where $z_{i}$ represents the *i*-th element of the vector *z*, and *N* is the number of classes. The output probabilities for all classes sum to 1.

## 4. Convolutional Neural Network Models

Various CNN architectures exist, each playing a vital role in developing deep learning models. Deep learning architectures have revolutionised prostate cancer diagnosis by automatically extracting complex features from imaging data. Early architectures like AlexNet and VGG laid the groundwork, while Inception introduced multi-scale feature extraction through parallel convolutions, improving computational efficiency and accuracy. This was followed by deeper and more efficient models such as ResNet, DenseNet, and Xception, which enhanced feature reuse and representation learning. Attention mechanisms like CBAM, residual attention neural networks, and ProstAttention-Net further advanced the models’ ability to selectively focus on clinically significant regions within prostate tissues, improving lesion localisation and classification. The introduction of U-Net-based architectures, including advanced variants like CSWin U-Net, combined with convolutional and transformer-based attention, to better capture both local and global contextual features, is crucial for precise segmentation tasks. More recently, comprehensive prostate cancer detection models that integrate multimodal data and sophisticated attention-based mechanisms have emerged, offering highly personalised and precise clinical decision-making capabilities that are increasingly approaching real-world clinical deployment. The following outlines the CNN architectures, and an overview of the strengths and limitations of each architecture is provided in Table 3 below.

### 4.1. AlexNet

The origin of deep learning models can be traced back to the introduction of LeNet [64], which, at the time, had limitations in recognising handwritten digits and did not apply to all types of images. Krizhevsky et al. [65] later introduced AlexNet, which enhanced the learning capability of CNNs by increasing their depth and applying various parameter optimisation techniques. Among deep CNNs architectures, AlexNet gained recognition for its performance in image recognition and classification [66]. Explainability is important for the clinician adoption of deep learning models like AlexNet. By integrating methods such as Grad CAM to visually highlight regions within prostate images that influenced the network’s predictions, clinicians can better understand, verify, and trust AI outputs, as these visual explanations allow direct comparison with their manual inspection process, reduce concerns over hidden biases, and foster confidence that the model is focusing on clinically relevant features rather than spurious image artifacts or noise. This integration significantly increases the acceptability of AI-assisted prostate cancer diagnosis among skeptical practitioners [67,68].

However, while hardware limitations still restricted the learning capacity of deep CNNs, this challenge was addressed by simultaneously using two NVIDIA GTX 580 GPUs which was manufactured by NVIDIA Corporation, based in Santa Clara, United States to train the model. To enhance CNN effectiveness across different image types, the number of feature extraction stages increased from five in LeNet to seven in AlexNet. AlexNet remains highly influential in modern CNN models, marking the beginning of a new era in CNN research. Researchers have adapted and refined AlexNet to analyse medical images such as MRI scans and histopathology slides for prostate cancer detection, showcasing its exceptional image-classification capabilities [65]. While ResNet and GoogLeNet offer improved performance compared to AlexNet, they demand more computational resources [69]. However, this model has significant trade-offs: Its large parameter size increases computational and memory demands, making training and deployment on limited resource clinical environments challenging. Compared to newer architectures, it is prone to overfitting smaller medical datasets common in clinical practice. Despite these limitations, AlexNet remains clinically relevant as a foundation for transfer learning, enabling rapid adaptation to specific medical imaging tasks with limited labelled data, thereby supporting diagnostic decision-making and early detection, and aiding radiologists in complex pattern recognition. The diagram in Figure 3 shows the basic structure of the model:

### 4.2. Visual Geometry Group (VGG) Network

After the success of convolutional neural networks in image detection, Simonyan and Zisserman proposed a simple yet effective design principle for CNNs [70], which was named VGG. The clarity of VGG networks stems from their straightforward, fully convolutional architecture with uniform small 3×3 filters and sequential layer stacking, which allows for easier visualisation and understanding of learned feature maps, thereby facilitating clinical trust and making them more interpretable compared to more complex architectures in prostate cancer diagnosis [70].

The innovative design introduced a model with multiple layers [71], incorporating 19 additional layers compared to ZefNet [72] and AlexNet [73], thereby increasing the network’s depth. VGG manages network complexity by using 1×1 convolutions between convolutional layers, which helps to linearly organise the feature maps. To optimise the network, a maximum pooling layer is added after each convolutional layer, with padding used to maintain spatial resolution [74]. VGG achieved impressive results in image classification and localisation tasks. It was also considered computationally expensive due to its reliance on approximately 140 million parameters, which was seen as its major drawback. However, the model has notable trade-offs. Its large number of parameters (especially in deeper variants like VGG-16 or VGG-19) leads to high computational and memory demands, making training and deployment resource-intensive. This can limit its real-time clinical applicability unless sufficient hardware resources are available. Additionally, VGG models are prone to overfitting when trained on small medical datasets, often requiring extensive data augmentation or transfer learning to generalise well. Clinically, VGG’s ability to capture detailed features makes it valuable for tasks like tumour grading, segmentation, and lesion detection in histopathology and radiology images. When combined with transfer learning on pre-trained weights (e.g., ImageNet), VGG can adapt to medical imaging tasks even with limited data to enhance diagnostic accuracy. Nonetheless, its resource demands and susceptibility to overfitting highlight the need for careful model optimisation and validation before clinical deployment. The network structure is depicted in Figure 3.

### 4.3. Inception

Inception, also known as GoogleNet, emerged as the winner of the 2014 ILSVRC competition [75]. The architecture was designed to achieve high accuracy while minimising computational costs. In clinical prostate imaging, especially for tasks like prostate lesion detection, explainability is vital because many clinicians remain cautious of using AI systems that function as `black boxes.’ The Inception network, with its multi-scale convolutional architecture, offers a certain degree of clarity by enabling the visualisation of feature maps at different receptive fields, which can correspond to anatomical structures and pathological features clinicians are familiar with. This transparency helps bridge the cognitive gap between algorithmic decision-making and expert clinical judgment, fostering greater trust and willingness among clinicians to adopt AI-assisted tools in routine prostate cancer diagnosis [76,77].

This model introduced an innovative inception block (or module) within the context of CNNs, combining merge, transform, and split operations to integrate convolutional transformations at multiple scales, thereby facilitating effective feature extraction. The design incorporates filters of various sizes, such as 5×5, 3×3, and 1×1 to capture spatial and channel information at different resolutions. Unlike traditional convolutional layers, GoogLeNet employs smaller blocks based on the network-in-network (NIN) architecture, where each layer functions as a mini neural network. The merge, transform, and split concepts address challenges associated with learning variations within the same image class. GoogleNet aims to optimise CNN parameters and enhance learning capacity to control computational demands, which includes a 1×1 convolutional filter and acts as a bottleneck layer before larger kernels and utilises sparse connections to reduce redundant information. It then links specific input channels to their respective output channels. Using a global average pooling (GAP) layer instead of a fully connected (FC) layer in the final stage significantly reduced the number of parameters from 40 million to just 5 million.

Additional refinements include using root mean square propagation (RMSProp) as an optimiser and batch normalisation to improve regularisation [78].

The model aimed to optimise CNN parameters and enhance introduced auxiliary learners to speed up convergence, but despite its strengths, it faces some drawbacks. Its intricate complexity demands precise tuning between modules and bottleneck layers. Although efficient, this design can sometimes excessively reduce the feature space, potentially leading to the loss of important information. The model offers strong multi-scale feature extraction leading to high accuracy in the diagnosis of prostate cancer, but its computational complexity and need for large annotated datasets present trade-offs, while its ability to improve diagnostic consistency and support complex grading tasks highlights its clinical relevance [33]. The architecture is shown in Figure 3.

### 4.4. Residual Neural Network (ResNet)

He et al. [57] developed ResNet, which won the ILSVRC 2015 competition. They intended to design an intense network capable of overcoming the vanishing gradient problem observed in earlier architectures. ResNet was introduced in several variants based on the number of layers, ranging from 34 to 1202 layers. Among the variants of this model, ResNet50 became the most widely used, having 49 convolutional layers and one fully connected (FC) layer. The integration of explainability into residual neural networks (ResNets) is important for clinical acceptance, particularly in prostate imaging, where clinicians have long relied on manual inspection and expert interpretation of gland morphology. While ResNets excel at capturing hierarchical features through skip connections and deep layers, their “black-box” nature can hinder clinician trust. By incorporating explainable AI (XAI) techniques such as saliency maps, Grad-CAM, or attention visualisation, ResNets can detect which regions or features contribute most to a model’s decision, allowing clinicians to verify that the AI model focuses on clinically relevant areas like peripheral zone lesions or suspicious nodules. This transparency fosters trust, facilitates clinical validation, and encourages adoption in real-world diagnostic workflows where accountability and interpretability are important [79,80].

**Figure 4:**
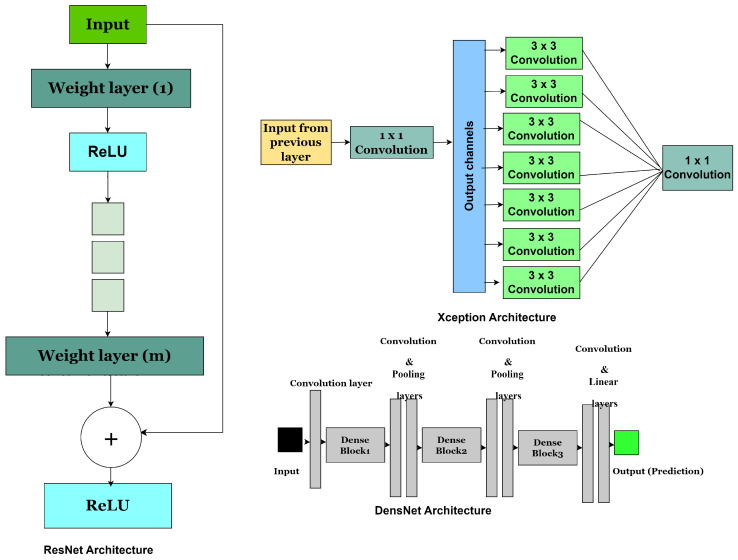
CNN architetures.

It has 25.5 million parameters and requires 3.9 million multiply–accumulate operations (MACs). The core of ResNet lies in its fundamental building blocks, known as residual blocks. The operations within these blocks were explicitly adapted to align with the overall structure of the network [57]. A distinguishing feature of ResNet is its use of shortcut connections that directly link layers. These connections, independent of the data and requiring no additional parameters, facilitate cross-layer connectivity. When the shortcut is inactive, the layers perform non-residual functions, like those found in highway networks. However, ResNet consistently maintains distinct pathways, ensuring that residual data are transmitted without interruption. By incorporating shortcut connections (residual links), ResNet effectively mitigates the vanishing gradient problem, enabling faster convergence in deep networks. It is significantly 20 times deeper than AlexNet and 8 times deeper than VGG while maintaining lower computational complexity than VGG despite having more layers. Variants of ResNet include models with 50, 101, and 152 layers. ResNet consistently achieves high classification accuracy and robust segmentation results across diverse datasets, and in prostate cancer applications, it has been successfully applied for Gleason grading, lesion detection, and MRI-based segmentation, often outperforming conventional CNN architectures. However, its deep architecture entails high computational cost, potential overfitting on small datasets, and limited interpretability that challenges clinical validation, though its capacity to capture subtle imaging features enhances early detection, risk stratification, and treatment planning across various modalities, provided issues of interpretability and reproducibility are adequately addressed for clinical integration [81]. The network structure is depicted in Figure 4.

### 4.5. DenseNet

This model, just like ResNet, was designed to tackle the problem of vanishing gradients. Unlike ResNet, which retains information through additive identity transformations that increase complexity, DenseNet addresses this issue by utilising inter-layer connections.The explainability of DenseNet models refers to methods that make the model’s decision-making process more transparent, such as generating heatmaps (e.g., Grad-CAM) that highlight which areas of the prostate the model focused on when making its prediction. These visual explanations help clinicians compare the AI’s reasoning with their observations, making it easier to trust and adopt the technology in clinical practice. Thus, improving explainability increases the acceptability of DenseNet models among clinicians because it allows them to confirm AI decisions, cross-check with their manual assessments, and feel more confident integrating AI support into their diagnostic workflows [80].

DenseNet employs dense blocks, where the feature maps from earlier layers are directly passed as inputs to subsequent layers, enhancing information flow and network efficiency [82]. As a result, this model outperforms traditional convolutional neural networks (CNNs) in tasks such as tumour segmentation, histopathological grading, and lesion classification, offering improved sensitivity and specificity [82]. Despite these advantages, DenseNet’s dense inter-layer connections increase computational complexity and memory demands during training, causing challenges for deployment in clinical settings with limited computational infrastructure. Moreover, the increased architectural complexity may hinder model interpretability, which remains a key concern in clinical practice where explainability is necessary to support diagnostic confidence and regulatory compliance.

Clinically, this model holds substantial promise in enhancing diagnostic accuracy by effectively capturing morphological heterogeneity and tissue texture variations present in prostate cancer. Its application can aid clinicians in early cancer detection, precise grading, and treatment planning, ultimately contributing to personalised patient care [83]. The architecture is shown in Figure 4.

### 4.6. Xception Architecture

In 2015, François Chollet, an artificial intelligence researcher at Google and creator of the widely used Keras Python API version 0.1.0 for neural networks, introduced a groundbreaking deep neural network architecture called the Xception network. The model was built on depth-wise separable convolutions [84]. Xception enhanced the Inception block by expanding its width and replacing the (3×3) and (1×1) convolution sequence with a more efficient design. Due to fine-grained feature extraction of this model, it has strong potential for prostate lesion detection, but its clinical acceptance critically depends on integrating explainability methods that allow clinicians to visually verify AI decisions, thereby fostering trust and adoption alongside manual inspection [17,85].

This model achieves greater computational effectiveness by decoupling channel and spatial correspondence. At first, it maps the convolved output to a reduced dimensionality using (1×1) convolutions, followed by k spatial transformations, where k refers to the cardinality, which determines the network’s width based on the number of transformations. Computations are streamlined by independently convolving each channel along the spatial axes, which are then processed with (1×1) convolutions, known as pointwise convolutions, to establish cross-channel relationships.

The (1×1) convolution in Xception is also employed to control channel depth. While traditional convolutional neural networks (CNNs) use a single transformation per layer, the Inception model uses three transformations, and the Xception design utilises transformations equal to the number of channels, offering more flexibility. Furthermore, the proposed modifications to Xception enhance learning efficiency and performance without increasing the parameter count [86]. Its ability to model complex spatial patterns allows it to achieve superior accuracy particularly in medical image analysis tasks where fine-grained texture and subtle differences are critical, such as differentiating cancerous from non-cancerous tissues in histopathological and radiological prostate images. However, the model requires large high-quality datasets to fully exploit its capacity, making it sensitive to data scarcity, a common limitation in specialised clinical domains like prostate cancer.

Also, while it reduces parameter count and computational load, it still demands considerable memory and GPU resources during training, which may not be ideal for all clinical environments [18]. Clinically, the model’s strong feature extraction makes it special for tasks such as the grading of Gleason patterns and lesion segmentation and feature extraction in MRI. Its precision supports improved diagnostic accuracy, potentially aiding radiologists and pathologists by providing consistent and reproducible assessments. The model adoption requires careful validation to ensure model generalisability across diverse patient populations and imaging protocols. The architecture is shown in Figure 4.

### 4.7. Convolutional Block Attention Module (CBAM)

This is a lightweight and efficient module designed to improve the feature representation capabilities of convolutional neural networks (CNNs). Introduced by Woo et al. [87], it leverages attention mechanisms along channel and spatial dimensions to highlight essential features while suppressing irrelevant ones in convolutional outputs. Despite the growing performance of AI models in prostate imaging, many clinicians still trust manual inspection because of its transparency and familiarity. The convolutional block attention module (CBAM) contributes to bridging this gap by providing attention maps that visually highlight which spatial regions and feature channels influenced the model’s predictions. This added level of explainability can help clinicians cross-reference AI outputs with their expertise, improving confidence in automated decisions and potentially increasing clinical acceptance [88].

The CBAM has demonstrated its effectiveness in enhancing the performance of deep learning models, especially in tasks such as image classification, object detection, and segmentation, where recognising subtle details is crucial [89]. The model also demonstrates enhanced sensitivity and Dice scores in image segmentation by refining feature maps to suppress irrelevant background noise and amplify lesion-specific signals, outperforming baseline architectures like vanilla ResNet or U-Net. These benefits come with trade-offs such as increased computational complexity, higher memory usage, risk of overfitting in small datasets, and slightly longer inference times. However, clinically, CBAM enhances interpretability and precision by highlighting diagnostically relevant regions, thereby supporting explainable AI and offering valuable assistance in decision support systems for prostate MRI and histopathological image analysis.

### 4.8. Residual Attention Neural Network

Wang et al. [90] introduced a residual attention network (RAN) that enhances the feature representation capabilities of convolutional neural networks (CNNs) by integrating attention modules. The model design allows the network to learn object-aware features effectively. The interpretability of this model, achieved through attention maps that visually highlight important regions influencing the model’s decisions, is vital for building confidence among clinicians who are traditionally reliant on manual inspection of prostate MRI. By offering transparent and intuitive visual cues that align with clinical reasoning, RANs help bridge the gap between automated AI predictions and human expertise, thereby increasing the possibility of clinical acceptance and integration into routine diagnostic workflows [91].

The architecture uses a feed-forward CNN composed of stacked residual blocks combined with attention modules. It integrates two complementary learning plans within the attention module, enabling fast feed-forward processing and top-down attention feedback in a unified process. This approach generates dense feature maps, facilitating pixel-wise inference. The bottom-up feed-forward structure generates low-resolution feature maps that are rich in semantic information. At the same time, the top-down strategy optimises the network globally, progressively refining feature maps throughout the learning process. The RAN was developed to address challenges in complex visual tasks, particularly those that require fine-grained detail recognition, such as image object detection, classification, and medical image analysis. This model offers a powerful framework for prostate cancer diagnosis by integrating residual learning with attention mechanisms, enabling the model to focus on clinically significant regions while maintaining stable and efficient training. This approach has shown superior performance in segmentation and classification tasks, particularly in detecting subtle lesions and achieving higher sensitivity and accuracy compared to conventional CNNs. However, the enhanced complexity introduced by attention modules demands greater computational resources and careful parameter tuning, which may limit scalability in resource-constrained clinical settings. Despite these challenges, the model’s ability to highlight diagnostically important areas enhances its clinical utility, supporting radiologists and pathologists in making more accurate and confident decisions, especially for early-stage or borderline cases, thereby contributing to improved patient outcomes [92].

This model equation can be expressed as follows: For input $X_{0}$ sent through *N* residual attention blocks, the output $Y_{N}$ of the residual attention network can be written as:

- The input feature map is *X*
- The weights of the residual function are F
- Residual mapping is F(X,W)
- Attention mask generated from the input *X* with weights $W_{A}$ is $A(X,W_{A})$.
- The output feature map after the attention mechanism and residual is applied is *Y*.
- Element-wise multiplication between the attention mask *A* and the residual mapping is F(X,W).

(3) $$Y_{\left(N\right)}=X_{\left(0\right)}+\sum_{i=1}^{N}\left(F_{i}\left(X_{\left(i-1\right)}\right)W_{i}\right)\cdot A_{i}\left(X_{\left(i-1\right)},W_{\left(A_{i}\right)}\right)$$

where

- $X_{\left(i-1\right)}$ represents the input to the *i*-th residual attention block.
- $F_{i}$ and $A_{i}$ represent the attention mechanism and residual function of the *i*-th block.

### 4.9. Transformer-Based Networks

In recent years, transformer-based networks have emerged as revolutionary architecture in deep learning, initially transforming natural language processing (NLP) tasks with models like BERT and GPT. Their application has now extended to computer vision, where vision transformers (ViTs) are becoming increasingly popular and are utilised in medical imaging [93]. For clinicians who still rely heavily on visually inspecting prostate images, the explainability of transformer-based networks is important because these models can highlight which parts of the image influenced their decisions, making it easier for doctors to understand, trust, and verify the AI’s findings, which helps encourage clinical adoption [94].

Adopting transformer-based networks in prostate cancer detection and classification marks a significant shift, offering distinct advantages over traditional convolutional neural networks (CNNs). This model utilises self-attention mechanisms to capture global dependencies in data, a feature that proves particularly beneficial for prostate cancer detection and classification. One key application of this model in prostate cancer is in classification and Gleason grading, where it has been used to analyse whole slide images (WSIs) and MRI scans to classify cancer grades using the Gleason grading system with remarkable accuracy. Chaurasia et al. [95] demonstrated, in their study, that the ViT-based model is highly effective in analysing histological images for diagnosing and grading prostate cancer. They found that it reliably distinguishes between benign and malignant tissues and can accurately classify malignancies based on their grade. Transformer-based networks such as vision transformers (ViTs) and their variants outperform CNNs on tasks like prostate gland segmentation and cancer grading by effectively capturing global context and long-range dependencies. However, they require large datasets, extensive computational resources, and longer training times compared to traditional CNNs [96]. Clinically, their ability to integrate multi-scale and multi-modal information (e.g., T2 weighted MRI, diffusion maps, and histopathology) makes them especially promising for personalised diagnosis, tumour delineation, and treatment planning [97].

### 4.10. CSWin U-Net

ProstAttention Net CSWin U-Net is a deep learning model that integrates the power of convolutional neural networks (CNNs) with cross-shaped window (CSWin) transformers to improve medical image segmentation [98]. Traditional U-Net architectures have been highly successful in biomedical image segmentation due to their encoder–decoder structure with skip connections, but they often struggle to capture global contextual information. CSWin U-Net addresses this limitation by embedding transformer-based self-attention modules into U-Net’s framework, enabling better global feature modeling. Explainability acts as a critical mediator between highly capable AI models like CSWin U-Net and the clinician’s trust, making its adoption feasible in real-world prostate cancer diagnosis workflows where manual inspection remains the gold standard [98].

### 4.11. ProstAttention Net

ProstAttention Net is a specialised model designed for the detection and classification of prostate cancer using medical imaging data, particularly histopathology or multiparametric MRI (mpMRI) data. The model integrates attention mechanisms into its architecture to improve the focus on diagnostically significant regions while minimising irrelevant information, thereby enhancing the accuracy and robustness of prostate cancer detection [99].

### 4.12. Prostate Cancer Detection Model (PCDM)

This deep learning model uses ResNet50 for deep feature extraction and Faster R-CNN for accurate lesion identification on prostate MRI images. ResNet50 captures both low-level and high-level imaging features, while Faster R-CNN’s region proposal network identifies and classifies suspicious lesions. This hybrid approach enables the simultaneous detection and classification of prostate cancer, improving diagnostic accuracy. The model demonstrated excellent performance with a sensitivity of 97.4%, specificity of 97.1%, and overall accuracy of 95.2%. Its end-to-end design assists radiologists in detecting clinically significant prostate cancer (csPCa), potentially reducing missed diagnoses. By leveraging transfer learning, the PCDM achieves high performance even with limited annotated data. However, its reliance on high-quality MRI data and bounding-box-based localisation may limit its ability to delineate precise tumour boundaries [100].

### 4.13. EfficientNet-B4 + Efficient Channel Attention (Eff4-Attn)

The Eff4-Attn model combines the EfficientNet-B4 convolutional neural network with the efficient channel attention (ECA) mechanism to create a scalable and efficient deep learning framework for prostate cancer classification using histopathological whole-slide images. EfficientNet-B4, known for its compound scaling of depth, width, and resolution, serves as a powerful backbone, pre-trained on ImageNet and fine-tuned on high-resolution pathology data to ensure strong baseline performance. The ECA module adds a lightweight yet effective channel-wise attention mechanism that enhances the model’s ability to focus on clinically relevant features without significantly increasing computational cost. This integration achieves an excellent trade-off between accuracy and efficiency, though the model’s complexity may present challenges for real-time use in low-resource settings [101]. Clinically, this model has shown high accuracy in critical diagnostic tasks such as Gleason grading, distinguishing cancerous from benign tissue, and assessing tumour aggressiveness, supporting pathologists, reducing manual workload, and enabling faster diagnoses. Its high speed and accuracy also make it suitable for telepathology and digital pathology workflows, particularly in regions with limited access to expert review [102].

### 4.14. Prostate Vision-Based Classification Network (ProViCNet)

This model is a 3D deep convolutional neural network designed to automatically classify clinically significant prostate cancer (csPCa) using multiparametric MRI (mpMRI). It processes three key MRI modalities, T2-weighted (T2W) imaging, apparent diffusion coefficient (ADC) imaging, and diffusion-weighted imaging (DWI), by combining them into a unified 3D feature space that captures both anatomical and functional information. The model uses a dual-branch architecture: Each modality is first processed through its dedicated convolutional path to extract unique features, then a fusion module combines the outputs to leverage complementary information [103]. An attention mechanism further enhances the model’s focus on critical tumour regions, especially in the transition and peripheral zones of the prostate, leading to improved classification among benign, clinically insignificant, and clinically significant lesions. This approach holds promise for reducing diagnostic variability among radiologists and increasing confidence in clinical decision-making. ProViCNet achieves high classification accuracy due to its multi-modality integration and attention-guided design. However, it depends heavily on high-quality mpMRI data. Its performance can drop with imaging artefacts, lower resolution, or missing modalities. Additionally, its 3D convolutional architecture is computationally demanding, requiring powerful GPUs and longer inference times, which may limit its use in clinics with limited computing resources.

### 4.15. Automated Multi-Modal Transformer Network (AMTNet)

This is a cutting-edge deep learning model developed for the segmentation and analysis of three-dimensional (3D) medical images, with proven effectiveness in prostate cancer detection. It features a dual-stream encoder architecture that processes different imaging modalities such as T2-weighted (T2W) MRI, diffusion-weighted imaging (DWI), and apparent diffusion coefficient (ADC) maps independently, preserving the unique characteristics of each modality [104]. These modality-specific features are then fused through a transformer-based module that uses self-attention mechanisms to capture long-range spatial relationships and complex interactions across modalities. By combining the local feature extraction strength of convolutional neural networks (CNNs) with the contextual modeling power of transformers, AMTNet significantly improves lesion localisation and anatomical segmentation. Its automated architecture reduces the need for extensive manual annotation and shows strong generalisability across varied clinical datasets, making it a valuable tool for accurate and efficient prostate cancer analysis. Despite its high segmentation accuracy and robust multi-modal integration, this model has certain trade-offs, including increased computational demands and longer training times due to its transformer components. However, its ability to model complex spatial dependencies and integrate diverse imaging sources makes it highly suitable for clinical applications, particularly in challenging or ambiguous diagnostic cases [104].

**Table 3:** Overview of CNN architectures: strengths and limitations.

Architectures	Description	Strengths	Limitations	Performance
ResNet [57]	A deep neural network architecture that revolutionised deep learning by enabling extremely deep networks to be trained effectively. It uses “skip connections” or “shortcut connections” to bypass one or more layers.	Prevention of vanishing gradients, increased depth and performance, a modular design for transfer learning, and parameter efficiency.	Computational intensity, complexity in network design, and dimensionality issues are key challenges.	3.57% error on imageNet test
AlexNet [65]	The model is made up of eight layers, including five (5) convolutional layers and three (3) fully connected layers, with ReLU activation functions used for non-linearity.	It offers performance improvement, efficient training with GPUs, the introduction of ReLU activation, and dropout regularization, though it is not robust for complex structures.	Large parameter count, susceptibility to overfitting, and inflexible architecture.	top-1 and top-5 error rates of 37.5% and 17.0%
VGG [70]	This model is known for its simplicity and depth. VGG network series (e.g., VGG16 and VGG19) uses 16 and 19 weight layers, respectively, consisting of multiple convolutional and fully connected layers.	Simplicity and modularity, deep feature hierarchy, and transfer learning are key principles.	High computational cost and memory usage, redundant parameters, and slow training time are key challenges.	top-1 and top-5 error rates of 23.7% and 6.8%
Inception [78]	This deep learning architecture revolutionised convolutional neural networks by introducing multi-scale processing within a single layer. The first model, Inception V1 (also known as GoogLeNet), uses an “Inception module” that applies multiple convolutions (1 × 1, 3 × 3, and 5 × 5) and pooling operations in parallel within the same layer.	The approach offers parameter efficiency, enables multi-scale feature extraction, adapts well to deep networks, and is effective for transfer learning.	Architecture complexity, computational demands, and higher memory requirements challenge the design of efficient and scalable deep networks.	top-1 of 21.2% and top-5 of 5.6% error
DenseNet [82]	A deep learning model that addresses some limitations of traditional CNNs by connecting each of the layers to other layers within a dense block, unlike typical CNNs, where each layer has its own set of filters and receives input from the previous layer. The model layers receive inputs from all preceding layers within each dense block.	Efficient parameter use enables improved gradient flow, promotes feature reuse and enhanced representation, and helps reduce overfitting.	Memory-intensive processes, increased computational complexity, and difficulty in fine-tuning	3.46% on C10+ and 17.18% on C100+ error rates
Xception [84]	It builds upon the Inception architecture but takes the concept of depthwise separable convolutions to an extreme. Standard convolutions are replaced with depthwise separable convolutions, where each channel of the input is processed independently before a pointwise convolution (1x1 convolution) is applied features.	Parameter efficiency and computational speed, enhanced feature extraction, and competitive accuracy.	Memory consumption, limited flexibility for transfer learning, and complexity in implementation are the primary challenges.	top-1 0.790 and top-5 0.945 accuracy
Convolutional Block Attention Module [87]	A lightweight attention model that enhances the feature learning ability of convolutional neural networks. It refines feature maps by applying two types of attention sequentially: channel attention and spatial attention.	Enhanced feature representation, lightweight efficiency, modularity, flexibility, and performance gains are achieved.	The limitations include sequential processing, limited adaptability to diverse data, and potential redundancy in simple architectures.	top-1 error of 29.27% and top-5 error of 10.09%
Residual Attention Neural Network [90]	It combines residual learning (from ResNet) with a self-attention mechanism to enhance feature extraction, which allows the network to focus on important parts of an image while ignoring irrelevant details. The network has a stacked structure of attention modules that perform spatial attention to highlight specific regions in the image and channel attention to emphasise useful features.	It enables selective feature learning, effective gradient flow, and modular attention blocks, while enhancing interpretability and proving useful in applications that require detailed feature focus, such as object detection, medical imaging, and facial recognition.	Increased computational complexity during training and a heightened risk of overfitting on small datasets.	top-1 accuracy 0.6%
Transformer-Based Networks [93]	It uses self-attention mechanisms to capture long-range dependencies and global contextual relationships within data, making them highly effective for complex tasks in both natural language processing and medical imaging.	Transformer-based networks are good at capturing global contextual information and long-range dependencies, leading to improved performance in complex data analysis tasks such as medical imaging and prostate lesion detection.	They require large amounts of data and significant computational resources for training and may struggle with limited datasets or real-time clinical deployment.	It achieved a κ score of 0.967
CSwin UNet [98]	CSWin U-Net is a hybrid deep learning model that integrates the U-Net architecture with the cross-shaped window transformer (CSWin transformer). The model leverages both convolutional neural networks (CNNs) and transformer-based self-attention mechanisms to enhance medical image segmentation, especially in complex tasks such as prostate cancer detection, brain tumour segmentation, and other biomedical imaging applications.	It combines the powerful global context modeling of the CSWin transformer with U-Net’s multi-scale feature extraction, enabling efficient long-range dependency capture, fine-grained segmentation accuracy, improved boundary detection, and reduced computational cost compared to full self-attention models, making it highly effective for complex medical imaging tasks.	Despite its strengths, this model remains more computationally intensive than traditional CNN-based models, requires large annotated datasets, is sensitive to hyperparameter tuning, may face generalisation challenges on unseen data, and typically demands longer training times.	It achieved 85.4% top-1 accuracy on ImageNet-1K and on ImageNet-21K it achieved 87.5% top-1 accuracy on ImageNet-1K
ProstAttention Net [99]	This model is developed for prostate cancer detection and makes use of attention mechanisms. It selectively focuses on important regions within the prostate, improving its ability to detect small or subtle cancerous lesions. The integration of attention modules enhances both accuracy and interpretability, making it a promising tool to support clinical decision-making.	It leverages attention mechanisms to focus on critical regions in prostate images, improving the detection of small or subtle cancerous lesions. Its attention maps offer some interpretability, making it more transparent for clinical use. The model performs well even with imbalanced datasets and is adaptable across different imaging modalities.	Added attention modules increase complexity, requiring more computational resources. It may risk overfitting, especially with limited labelled data. Despite the use of attention, full explainability remains limited, and its generalisability across different clinical settings may still be a challenge without extensive multicenter validation.	Dice of 0.875±0.013
PCDM model [100]	A hybrid deep learning approach that combines ResNet50 for extracting detailed imaging features and Faster R-CNN for accurately detecting and classifying prostate cancer lesions on MRI scans, achieving high sensitivity and specificity in identifying clinically significant tumours.	The PCDM model achieves highly accurate prostate cancer detection by combining powerful feature extraction from ResNet50 with precise lesion localisation from Faster R-CNN, resulting in excellent sensitivity, specificity, and diagnostic reliability.	Its reliance on bounding-box detection may limit fine-grained lesion boundary delineation, and its performance can be affected by variations in MRI quality and acquisition protocols across institutions.	Accuracy of 95.2%, sensitivity of 97.4% and specificity of 97.1%
Eff4-Attn [101]	A deep learning model that combines EfficientNet B4 with efficient channel attention (ECA) to achieve high accuracy and speed in image classification. Its balanced architecture enhances feature focus while remaining lightweight, making it ideal for medical imaging tasks like prostate cancer diagnosis in digital and telepathology settings.	This model delivers high diagnostic accuracy, operates efficiently with fewer parameters and robustly detects fine tissue features using channel-wise attention. It performs well across different magnifications and enhances interpretability, making it ideal for real-world pathology applications.	Despite its high performance, the model faces limitations such as sensitivity to training data quality, reliance on time-consuming patch-based WSI processing, and the need for clinical validation to ensure real-world applicability and regulatory approval.	Cancer detection accuracy of 96.18% and Gleason grade accuracy of 94.86%
ProViCNet [103]	A 3D deep learning framework developed for automated classification of clinically significant prostate cancer (csPCa) using multiparametric MRI (mpMRI). It integrates T2-weighted, ADC and DWI sequences into a unified 3D feature space, using a dual-branch architecture with modality-specific pathways and an attention mechanism to focus on critical prostate regions.	The model effectively integrates multiple mpMRI modalities with attention-guided feature extraction, leading to high accuracy in detecting clinically significant prostate cancer and reducing inter-reader variability.	Its performance depends on high-quality, complete mpMRI data, and its 3D architecture requires substantial computational resources, which may hinder deployment in low-resource clinical settings.	AUROC of 0.907 Sensitivity and Specificity of 0.880 and 0.654
AMTNet [104]	A deep learning model designed for 3D medical image segmentation, particularly in prostate cancer detection. It uses a dual-stream encoder to process multiple imaging modalities such as T2-weighted MRI, DWI, and ADC maps and fuses them through a transformer-based attention mechanism.	It effectively captures long-range spatial dependencies and integrates diverse imaging modalities, leading to highly accurate lesion localisation and segmentation in prostate cancer detection.	The model’s transformer-based architecture results in increased computational complexity and longer training times, which may limit its scalability in resource-constrained clinical settings.	Achieves an average DSC of 0.907 and 0.851

## 5. Deep Learning Applications for Image-Based Prostate Cancer Diagnosis

Applications of deep learning (DL) in medical images encompass image detection, classification, and segmentation, which are crucial tasks in medical image processing [105]. In applying deep learning to medical tasks, numerous curated datasets are used to train deep neural networks, yet data availability remains a significant challenge in the field.

However, transfer learning techniques have been employed to address the issue of data scarcity. Fine-tuning a pre-trained network and fixed feature extractors are two shared transfer learning methods. Deep learning models can classify images and are utilised in the image detection process due to their ability to accurately identify tumours and organs through medical image processing [106].

### 5.1. Magnetic Resonance Imaging for Prostate Cancer Diagnosis

According to a study conducted by [107], deep learning models were used to distinguish between cancerous and non-cancerous tumours, as well as high-risk and low-risk tumours. Magnetic resonance imaging datasets of 400 patients were detected as showing prostate cancer. The model obtained AUCs of 0.89 (95% CI: [0.86–0.92]) for differentiating cancer from non-cancer cases and 0.78 (95% CI: [0.74–0.82]) for differentiating high-risk from low-risk prostate disease. Also, a study by [108] used diffusion-weighted magnetic resonance imaging (DW-MRI) and mono-exponential function and dilated cardiomyopathy (DCM) using a deep learning model U-Net to examine a short-term test and then retest the accuracy of the U-Net in slice, in lesion level identification and segmentation significant of prostate cancer clinically (csPCa: Gleason grade group > 1) (U-Net). The dataset used for the training includes 112 prostate cancer MRI images of patients. Both CNNs and U-Net-based models achieved intraclass correlation coefficients between 0.80 to 0.83 percent agreement of 66–72 percent and DSC of 0.68 to 0.72 for slice and level of lesion identification. This research finding proved the strength of deep learning architecture in testing, retesting, and accuracy in detecting and segmenting clinically relevant prostate cancer on the apparent diffusion coefficient maps [108].

In the study by [109], the authors developed a computer-aided diagnostic system to detect prostate cancer early using diffusion-weighted magnetic resonance imaging (DW-MRI), which was collected at varying b-values. The proposed system consists of three primary stages. The initial phase involves prostate segmentation using a hybrid framework that combines a geometric deformable model, level sets, and non-negative matrix factorisation (NMF), followed by the estimation of the apparent diffusion coefficient (ADC) of the segmented prostate volume at various b-values and refinement with a generalised Gauss–Markov random field (GGMRF) image model.

Lastly, a two-stage stacked non-negativity constraint autoencoder (SNCAE) structure is used to classify prostate tumours as benign or malignant based on constructed cumulative distribution functions (CDFs), demonstrating the potential of the proposed CAD system as a dependable non-invasive diagnostic tool.

Hong et al. [70] examined the practicality of using a deep learning algorithm with apparent diffusion coefficient (ADC) maps to categorise and differentiate clinically significant cancer (CSC, Gleason score ≥7) from non-CSC in prostate cancer (PCa) patients. The dataset was obtained from 149 patients who had received 3T-MRI scans and were pathologically confirmed to have prostate cancer. One hundred and forty-eight images were labeled for GS6, while 580 images were labeled for GS7 and used for tumour segmentation with convolutional neural networks (CNNs). Ninety-three images were utilised for GS6 classification, while 372 images were used for GS7 classification. Twenty-two consecutive patients from five different institutions were recruited for external validation, comprising 25 images for GS6 and 70 images for GS7, all obtained from various magnetic resonance (MR) machines. The results obtained indicated that U-Net was utilised for segmentation, whereas DenseNet was employed for classification. The tumour Dice scores were 0.822 and 0.7776 for internal and external validation, respectively. Regarding classification, the internal and external validation accuracies were 73% and 75%, respectively.

The external validation showed CSC diagnostic predictive values of 84% sensitivity, 48% specificity, 82% positive predictive value, and 52% negative predictive value. The researchers concluded that it is possible to segment tumours and differentiate between CSC and non-CSC using a DLA created from ADC maps (b2000) only.

### 5.2. Deep Learning Model for Histopathological Diagnosis of Prostate Cancer

Researchers have also studied the application of deep learning in the detection of prostate cancer using histopathological diagnosis. In a study conducted by [110], the researchers automatically used a deep learning approach for pattern classification of the Gleason grade and identification of grade groups for prostate biopsies. A dataset of 96 prostate biopsies obtained from 38 patients was used in their study. However, the InceptionV3 CNNs model was trained to produce probability maps which then gave an accuracy of 92% to differentiate among non-atypical, malignant regions with 90% and 93% sensitivity and specificity, respectively. This study was successfully able to prove the possibility of using CNNs for training to distinguish between Gleason patients in heterogeneous biopsies [110].

Deep learning algorithms were developed by [111] to aid in the diagnosis of histopathology. In the study, Gleason grading of the cancer of the prostate biopsies was also performed. The process of diagnosing histopathology and grading the Gleason score was considered laborious; however, it was made quicker and more effective by using machine learning models. Eighty-five prostate biopsy images from 25 patients were used in the study, which included additional magnification up to 20x. A deep residual convolutional neural network (CNN) and fivefold cross-validation were employed in the study. At the coarse and fine-level classifications, the deep learning model obtained accuracies of 91.5% and 85.4%, respectively. The study found that, for the diagnosis described above, the model performed excellently, and it was concluded that the model needs to be evaluated on a large sample size for better justification of the performance [111]

### 5.3. MRI-Based Segmentation Techniques for Prostate Cancer Diagnosis

A study by [111] utilised an MRI of prostate cancer to compare the strength of a deep learning model with that of multiple radiologists’ experts. The MRI images used were obtained from 165 suspected patients with prostate cancer. The U-Net model was used to train the MRI dataset for segmentation operations. The mean Dice coefficient result obtained using manual segmentation was 0.48 to 0.52, while the U-Net model performed with a Dice coefficient (DE) of 0.22. The researchers concluded that the lower value of Dice coefficients in the U-Net framework was due to smaller segmentation sizes.

Additionally, a study conducted by [112] utilised deep learning (DL) to assess the speed and accuracy of prostate gland segmentation using MRI images. The researcher made use of images from 905 patients who had undergone prostate cancer MRI scans from 29 institutions. The ProGNet model was trained in 805 cases and subsequently tested using 100 independent internal and 56 external cases. Ultimately, this model outperformed the U-Net model. The study revealed that ProGNet required just 35 s per case, compared to the 10 minutes it took radiology technicians to produce a clinically usable segmentation file. The study stated that DL models could be utilised for target biopsy segmentation in everyday urological clinical practice [113].

In the study by [108], a DCNN model was developed for segmenting the prostate zone and identifying cancer regions in MRI images. A dataset of PROSTATEx, comprising MRI scan images of 204 patients, was utilised. The SegNet architecture was enhanced and subsequently optimised for improved performance. The concluding result of the research shows that the Dice similarity coefficient for the transition zone was 90.45%, for the peripheral zone, 70.04%; and for the region of prostate cancer, 52.73%. The study concluded that using DCNN for automatic segmentation has the potential to enhance prostate cancer diagnosis [108].

Schelb et al. [111], discovered that DL-based methods using a U-Net were effective in a study with 312 patients, yielding positive outcomes. A sensitivity and specificity of 96% and 88%, and 22% and 50%, were reported for radiologists using PIRADS to detect PIRADS lesions grade ≥3 and 4, In comparison, the U-Net approach showed 96% and 92% accuracy, as well as 31% and 47% sensitivity, respectively (p>0.05). The U-Net also automatically delineated the prostate and lesion in this research, with a Dice coefficient of 0.89 (excellent) and 0.35 (fair), respectively.

The researchers in [114] utilised the 3D-Mask RCNN model in their investigation for segmenting prostate tumours and identifying cancer through MRI T2WI, demonstrating high accuracy and efficiency. The technique employed could significantly aid radiologists by enhancing the precision and speed of diagnoses, leading to more effective treatment strategies for patients with prostate cancer.

The authors in [115] proposed a deep learning architecture, the SEMRCNN model, for the automatic segmentation of prostate cancer from multiparametric magnetic resonance image regions. The goal of their study was to use feature maps to achieve detailed segmentation within MRI image regions. Two convolutional networks were used in parallel to extract apparent diffusion coefficient (ADC) and T2W image maps, which were subsequently combined to leverage the complementary data in MP-MRI.

The model achieved a Dice coefficient of 0.654, a sensitivity of 0.695, a specificity of 0.970, and a positive predictive value of 0.685 for segmenting prostate cancer lesions. The model proves to be more effective than the Resnet50, V-Net, U-Net, and Mask-RCNN models in segmenting prostate cancer on MP-MRI. This method achieves precise segmentation of lesions by identifying and locating potential areas of prostate cancer (PCa) lesions.

### 5.4. Detection of Prostate Cancer with Computed Tomography Images

Several studies have utilised deep learning algorithms to detect prostate cancer and for applications in prostatectomy. DL has been used to automatically detect the quantity of prostate cancer on positron emission tomography and computed tomography (PET/CT) scans in research conducted by [113].

Additionally, a study performed by [116] utilised deep learning with multi-atlas fusion to achieve automatic prostate segmentation. Ninety-two prostate CT scans were used in the study, and their performance was compared with that of the radiologists’ traditional segmentation approach. The deep learning framework achieved an 86.80% Dice similarity coefficient. It was concluded that a deep learning-based approach can also make a significant contribution in automating segmentation, which will also benefit clinical practice [116].

In research conducted by [105], a non-invasive CAD system was introduced that combines PSA screening outcomes with DW-MRI-based characteristics to aid in the detection of prostate cancer. The CAD system demonstrated in this study combines level sets and NMF for prostate segmentation, creates global features from ADC-CDFs to distinguish between benign and malignant cases, and utilises two-phase SNCSAEs on ADC-CDFs and PSA-based probabilities for a more robust prostate cancer diagnosis. According to [117], the study employed a robust deep learning convolutional neural network (CNN) utilising a transfer learning technique. Different machine learning techniques, such as the SVM with other kernels, the decision tree, and Bayes, were used for the comparison of results. Table 4 summarises the state-of-the-art deep learning algorithms and their performance across the various studies described above.

## 6. Prostate Cancer Dataset

Prostate cancer datasets are essential tools for researchers and policymakers. They enable the collection of extensive, standardised data that can be examined to gain a deeper understanding of the disease’s epidemiology and treatment results, and aid in creating improved diagnostic and therapeutic methods. These databases facilitate collaboration among experts from various institutions on research projects focused on prostate cancer, enabling them to share their knowledge and resources. Here is a summary of databases related to prostate cancer:

### 6.1. Digital Pathology Dataset for Prostate Cancer Diagnosis [116]

Digitised hematoxylin and Eosin (H&E) stained whole slide images (WSis) with 59 core needle biopsy specimens and 40 prostatectomies were obtained from 99 prostate cancer individuals at the Tan Tock Seng Hospital in Singapore. The total number of WSIs was 99, each of which had one WSI. Hematoxylin and Eosin-stained slides were scanned at 40x magnification, with a specimen-level pixel size of 0.25 µm× 0.25 µm, while an Aperio AT2 slide scanner was used (Leica Biosystems). This dataset is used for training two artificial intelligence models: one for gland segmentation in 99 patients and another for gland classification in 46 patients.

### 6.2. Peso Dataset [120]

This dataset comprises 102 complete slide images, which were divided into training and testing sets. The dataset was collected from Radboud University Medical Centre at various points between 2006 and 2011, under IRB code 2016-2275. The training dataset includes 62 raw colour deconvolution masks, 62 training masks, 62 whole slide images (WSI), and 25 colour deconvolution masks. The testing dataset comprises 40 WSI and 40 XML files with 160 annotations, 160 PNG files for a test region, PNG files with padding for the same region, and a CSV file indicating the presence of cancer or tissue in the test area.

### 6.3. TCGA Prostate Adenocarcinoma Dataset (TCGA-PRAD) [121]

This dataset comprises 500 clinical cases, of which 490 patient cases are available. Biospecimens were collected from 500 cases. TCGA tissues were obtained from various locations worldwide to meet the target of approximately 500 specimens of different types of cancer. However, image datasets in the form of manufacturing, scanner modalities, and processing protocols were shown to be highly heterogeneous. Typically, the images were obtained during standard care rather than a controlled research study or clinical trial.

### 6.4. Gleason Challenge [122]

**Figure 5:**
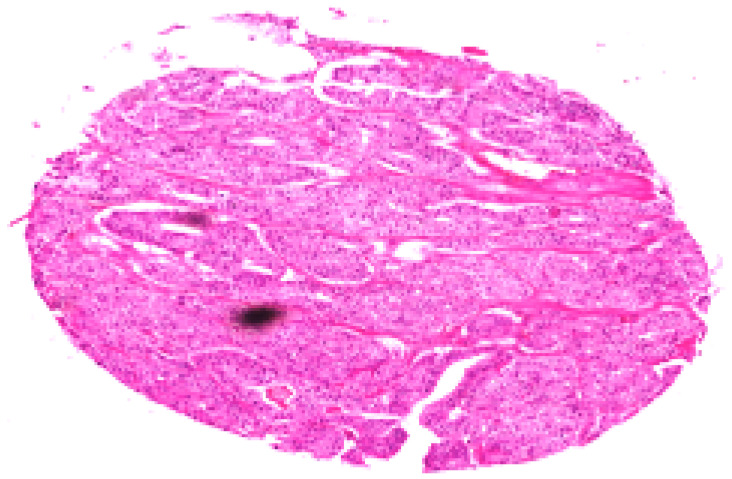
Gleason 2019 challenge image sample.

This dataset consists of the 2019 Gleason Challenge, which is a portion of the Pathology Challenge at the Medical Image Computing and Computer-Assisted Intervention (MICCAI) conference in 2019. It consists of a set of micro-arrays (TMA) images. The training dataset consists of approximately 244 prostate Tissue Microarray (TMA) images, while the test dataset comprises 87 prostate Tissue Microarray (TMA) images. The TMA images were obtained from the Vancouver Prostate Center. Every single Tissues Microarray image is meticulously tagged with a level of benign, Gleason grade 3, Gleason grade 4, and Gleason grade 5 by numerous experienced pathologists. An example of the 2019 Gleason challenge can be found in Figure 5:

### 6.5. Prostate Cancer Grade Assessment (PANDA) Challenge [123]

**Figure 6:**
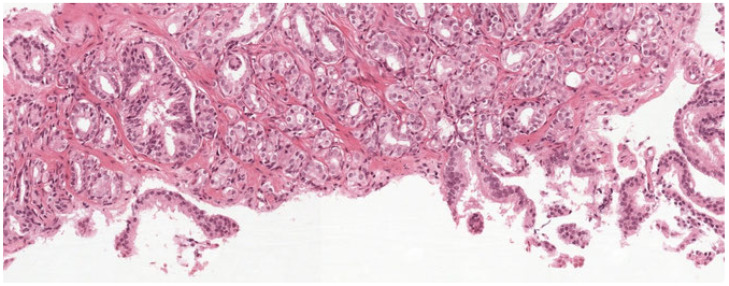
PANDA challenge sample [122].

PANDA remains the most extensive publicly available dataset of whole-slide images, which is approximately eight times the size of the CAMELYON17 challenge. This dataset was created by a team of computational pathologists from Radboud University Medical Center, in collaboration with the Department of Medical Epidemiology and Biostatistics at the Karolinska Institute. The training dataset contains approximately 11,000 whole-slide images of digitised H&E-stained biopsies [111]. All slides were scanned at 20× magnification and converted to TIFF format. A combined total of 800 whole slide images was included in both the public and private tests. One tissue specimen taken for biopsy corresponds to one specific case. The same patient may appear in multiple instances; however, patients in the test dataset are distinct from those in the training dataset, and label noise is present in the training dataset. Inaccurate pathologist findings, mistakes in annotations and initial diagnoses, and disagreements among pathologists all contribute to creating this label noise. A sample image of the panda challenge is shown in Figure 6.

### 6.6. PROSTATE-MRI [123]

This prostate MRI was acquired using a 3T MRI machine (Philips Achieva) with both endorectal and phased array surface coils which was manufactured by Philips Healthcare located in Best, Netherlands. Every patient received a biopsy confirmation of cancer and had a robotic-assisted radical prostatectomy. A mold was created from every MRI scan, with the prostatectomy specimen positioned in the mold before being cut along the same plane as the MRI image. The information was produced at the National Cancer Institute in Bethesda, Maryland, USA, from 2008 to 2010.

### 6.7. NCIGT-PROSTATE [124]

The National Center for Image-Guided Therapy-Prostate (NCIGT-PROSTATE) is a research program at the National Institutes of Health (NIH) that focuses on accelerating the diagnosis and treatment of prostate cancer using emerging image-guided therapies. As part of the broader mission of the National Center for Image-Guided Therapy (NCIGT), this activity aims explicitly to enhance the accuracy and efficiency of cancer and disease treatments using the latest imaging technologies.

A significant focus of NCIGT-PROSTATE is the refinement of the prostate biopsy procedure, which is of tremendous clinical importance for accurate diagnosis. For example, MRI-guided biopsies provide a more precise delivery of biopsy needles in comparison to conventional ultrasound-guided biopsies. The project further investigates minimally invasive focal therapies such as high-intensity focused ultrasound (HIFU), cryotherapy, and laser ablation, which serve as complements to conventional surgery with a reduced incidence of complications associated with incontinence and erectile dysfunction.

The core of the program is centered around multiparametric MRI (mpMRI), which combines anatomical, functional, and molecular imaging, providing a holistic picture of the prostate. This technology can enhance cancer detection, staging, and treatment decisions by differentially segmenting aggressive and indolent tumours, thereby avoiding unnecessary treatments. NCIGT-PROSTATE is a landmark breakthrough in the management of prostate cancers by emphasising individualised, accurate, and patient-oriented treatments. Advancements in imaging, robotics, and AI not only enable early and precise diagnosis but also pave the way for personalised and minimally invasive therapies tailored to meet each patient’s unique needs.

### 6.8. PROMISE12 Challenge [125]

The PROMISE12 Challenge aimed to improve the precision of automatic prostate gland segmentation in MRI through a scientific competition. Initiated in 2012, the competition challenged scientists and programmers to devise and evaluate algorithms capable of automatically and accurately delineating the prostate in MRI scans, a critical step in the diagnosis, staging, and planning of prostate cancer treatment. The challenge provided an example dataset of 50 prostate MR scans from patients, containing T2-weighted MR images and corresponding ground-truth segmentations created by the radiologist.

The PROMISE12 dataset and challenge framework still serve as a standard for researchers to develop and benchmark new prostate segmentation algorithms. The achievement of PROMISE12 has driven corresponding problems in other domains of medical imaging, driving the evolution of automated image analysis and computer-aided diagnosis.

### 6.9. PROSTATE158 [71]

Prostate158 is a publicly available dataset that is intended in the context of prostate cancer research to facilitate prostate segmentation and image-directed interventions. It consists of 158 MRI scans of patients with anatomical information on the prostate gland. Each scan comprises high-resolution T2-weighted images, which are one of the most used imaging sequences in prostate cancer diagnosis because they can create detailed images of the prostate and its surrounding tissues. This dataset has been applied to a wide range of tasks, including the training, testing, and validation of algorithms used for automated prostate segmentation on T2-weighted magnetic resonance imaging (MRI) scans, thereby facilitating the development of tools to aid in the diagnosis and treatment of prostate cancer. Researchers leverage Prostate158 to train deep learning models, which have become standard in medical imaging for segmentation tasks. These models are utilised in a wide range of applications, including clinical decision-making, prostate cancer screening, and treatment planning.

### 6.10. QIN-PROSTATE-Repeatability [126]

The QIN-PROSTATE-Repeatability study, one of the projects undertaken under the Quantitative Imaging Network (QIN), examines the reproducibility of quantitative imaging biomarkers in prostate cancer. Its main objective is to verify the consistency and reproducibility of imaging characteristics, particularly on T2-weighted MRI and diffusion-weighted imaging (DWI) across a series of scans. Establishing repeatability is crucial for developing robust imaging biomarkers that support prostate cancer detection, treatment planning, and monitoring. The study includes T2-weighted MRI, which provides detailed visualisation of prostate structures, and DWI, which evaluates tumour aggressiveness by measuring water molecule diffusion in tissues. An overview of key publicly available prostate cancer datasets and grand challenges are summarised in Table 5, which provides a concise comparison of their characteristics, sources, modalities, and applications.

## 7. Performance Metrics

Evaluation metrics in deep learning tasks play a crucial role in optimising the performance of classifiers [71]. These metrics are applied in the typical data categorisation process, which involves two main phases: training and testing. During the training phase, evaluation metrics refine the classification algorithm. This means the metric serves as a discriminator helping to identify and select the best solution to provide more accurate predictions for future evaluations of a specific classifier.

During the testing phase, the evaluation metric is used to assess the effectiveness of the trained classifier, which serves as a validator when the model is tested with unseen data [90]. The following equations define the metrics: TN and TP represent the number of negative and positive instances correctly classified. FN represents the number of positive cases misclassified, while FP indicates the number of negative instances misclassified. Below is a list of some of the most widely used assessment criteria.

### 7.1. Accuracy

Accuracy determines the proportion of accurately predicted classes from the total samples assessed.

(4) $$\mathrm{Accuracy}=\frac{\left|C\right|}{\mathrm{Total}\;\mathrm{Samples}}=\frac{\left|C\right|}{N}$$

where *C* is the set of correctly classified samples and *N* is the total number of samples.

### 7.2. Recall

This calculates the accuracy rate of correctly identifying positive patterns.

(5) $$\mathrm{Recall}=\frac{\mathrm{True}\;\mathrm{Positives}}{\mathrm{True}\;\mathrm{Positives}+\mathrm{False}\;\mathrm{Negatives}}=\frac{TP}{TP+FN}$$

### 7.3. Specificity

Specificity determines the percentage of negative patterns that are accurately classified, using the false positive rate (FPR):

(6) Specificity=1−FPR

where $\mathrm{FPR}=\frac{FP}{FP+TN}$

### 7.4. Precision

Precision determines how many of the predicted positive patterns are actually correct.

(7) $$\mathrm{Precision}=\frac{\mathrm{True}\;\mathrm{Positives}}{\mathrm{Predicted}\;\mathrm{Positives}}=\frac{TP}{TP+FP}$$

### 7.5. F1-Score

The F1-Score is the harmonic mean of precision and recall:

(8) $$F_{1}=\frac{2\times \mathrm{Precision}\times \mathrm{Recall}}{\mathrm{Precision}+\mathrm{Recall}}=\frac{2TP}{2TP+FP+FN}$$

### 7.6. False Positive Rate (FPR)

This metric calculates the probability of a false alarm:

(9) $$\mathrm{FPR}=1-\frac{TN}{FP+TN}$$

## 8. Discussion

This review presents an analysis of significant findings, explores the challenges associated with prostate lesion detection, and evaluates the strengths and limitations of variouse deep learning techniques. Identifying prostate lesions presents substantial challenges due to variations in image types and sources, which are discussed in this study. These variations result in inconsistencies and difficulties in accurately detecting lesions. Deep learning (DL) techniques have demonstrated outstanding potential in prostate lesion detection and analysis, particularly when utilising imaging modalities such as MRI, ultrasound, and histopathology. However, several obstacles persist in existing research, limiting the widespread clinical adoption of these methods.

A significant limitation is the reliance on small or private datasets, which restricts the capacity of generalising deep learning (DL) models. Although publicly available datasets, such as ProstateX and the PANDA challenge dataset, are valuable, they often lack diversity in patient demographics and variations in imaging scanners [122]. Small datasets increase the risk of overfitting, where models achieve high accuracy on training data but perform poorly on unseen data [99].

Annotating prostate lesion data is another challenge, as it is time-consuming and requires significant domain expertise. Interobserver variability in Gleason grading further complicates the creation of consistent annotations [128]. Additionally, deep learning models often encounter domain shifts resulting from variations in imaging protocols, scanner manufacturers, and institutions. This leads to reduced performance when models trained on one dataset are applied to data from another centre [129]. Integrating multiple modalities such as MRI, histopathology, and genomic data can improve model performance, but they also introduce added complexity. Their challenges include the misalignment of multimodal data, differences in resolution, and the difficulty of integrating data effectively [118].

Incorporating deep learning models into clinical workflows remains challenging due to issues such as software compatibility, regulatory requirements, and the need for clinician training. However, a practical pathway for including DL models in clinical workflows requires developing interoperable software that can integrate seamlessly with existing clinical systems and ensure compliance with regulatory standards through rigorous validation and implement comprehensive clinician training programs alongside explainable AI tools to build trust and promote adoption.

Future research should focus on the development of large diverse public datasets, advanced annotation tools, robust domain adaptation techniques, effective multimodal data fusion methods, and improved clinical integration. Addressing these areas will help overcome current limitations and support the safe, effective, and reliable clinical adoption of deep learning models for prostate cancer diagnosis. To overcome inconsistencies in image annotation and labelling across existing and future datasets, standardised annotation protocols should be developed and widely adopted, combining consensus guidelines like PI-RADS for prostate MRI and expert-reviewed labelling frameworks. The use of collaborative multi-reader annotations, supplemented by AI-assisted annotation tools, can reduce inter-observer variability and improve consistency. Furthermore, establishing centralised, publicly available benchmarking datasets with well-documented annotation procedures will facilitate reproducibility and fair model comparisons. Continuous training and certification programs for annotators can also ensure the consistent application of labelling standards over time, while semi-supervised and active learning approaches can reduce the manual annotation burden and promote high-quality label generation even as new datasets emerge.

## 9. Conclusions

This paper provides a critical and analytical overview of the state-of-the-art techniques for analysing prostate lesion images. It presents a comprehensive review of the methods and algorithms employed in image processing for detecting prostate cancer. The strategies discussed encompass pre-processing techniques, feature extraction methods, segmentation algorithms, and classification approaches. The study also examines both traditional techniques and the latest advancements in the field. The performance of state-of-the-art deep learning algorithms was critically analysed. As reviewed from the literature, deep learning models have performed well, especially in medical imaging prediction, segmentation, and classification. However, some of these models require significant computational resources and necessitate considerable training time.

It was observed that applying deep learning models to pre-processed and segmented images enhances classification performance across evaluation metrics. Pre-processing techniques, such as deep learning-based methods and intelligent segmentation approaches, have proven highly effective and are recommended for achieving better classification outcomes. These methods address the challenges of analysing prostate lesion images, which are characterised by their fine-grained appearance.

Lastly, a variety of prostate cancer datasets were reviewed. These datasets provide comprehensive and standardised data, enabling a deeper understanding of the disease’s epidemiology and treatment outcomes. These datasets also support the development of enhanced diagnostic and therapeutic approaches. Furthermore, they facilitate collaboration among experts from different institutions by promoting the sharing of knowledge and resources for prostate cancer research.

## Figures and Tables

**Table 1:** Comparison between segmentation techniques, highlighting their strengths and weaknesses.

Method	Description	Advantage	Disadvantage
Edge detection and contour features [45]	Handcrafted feature-based method. Examples include the Canny edge detector and active contour models (snakes).	The method offers simplicity and efficiency, enables sharp boundary detection and effective feature extraction, and reduces data complexity, and it is versatile across different modalities.	It is sensitive to noise, struggles with weak or fuzzy boundaries, produces fragmented contours, tends to over-segment, and lacks robustness for complex structures.
Intensity-based features [46]	Handcrafted feature-based method relying on pixel intensity values. Examples include thresholding and histogram analysis.	It offers simplicity and efficiency, fast processing with minimal user interaction, effective performance on high-contrast images, and versatility across different imaging modalities.	The method exhibits sensitivity to noise and artifacts, struggles with low-contrast images, is prone to over- or under-segmentation, is limited to simple structures, and is highly dependent on threshold settings.
Region-based features [47]	Divides an image into regions based on pixel similarity. Examples include region growing and the watershed algorithm.	It preserves spatial relationships and is effective for homogeneous regions, less sensitive to noise, robust under varying contrast, and capable of handling complex structures.	The method is prone to over-segmentation, sensitive to initialisation and intensity gradients, and computationally complex, and it struggles to handle heterogeneous regions.
Texture-based segmentation [48]	Analyzes pixel intensity patterns to differentiate image regions. Examples include GLCM and wavelet-based methods.	It captures fine details, performs effectively in heterogeneous regions, enables versatile feature extraction, improves segmentation of complex structures, and is resistant to illumination variations.	It suffers from high computational complexity, sensitivity to noise, difficulty in handling homogeneous regions, limited interpretability, and over-segmentation in high-texture areas.
Traditional intelligence-based methods [49]	Includes methods like ANN, genetic algorithms, fuzzy logic, and SVMs.	Interpretable models with low computational requirements, effective on small and less data-intensive datasets, and flexible across different problem types.	The approach suffers from limited accuracy, poor generalisation, reliance on manual feature engineering, susceptibility to noise, and difficulty handling multimodal data.
Deep learning-based methods [50]	Uses models like CNNs and U-Nets for automatic feature learning and segmentation.	Automatic feature learning, high accuracy, adaptability to complex structures, end-to-end learning, and robustness to noise and variability.	The method involves high data requirements, significant computational cost, risk of overfitting, extensive preprocessing, and a time-consuming training process.

**Table 2:** Comparison of traditional techniques commonly employed for classifying prostate lesions and state-of-the-art methods.

Methods	Outline	Strengths	Limitations	Applications
Traditional intelligence-based methods [19]	Relies on structured rules and algorithms for reasoning and classification.	Low data requirements and interpretability.	Limited flexibility and scalability challenges.	Expert systems and financial analysis.
Deep Neural Networks [60]	A neural network model with multiple layers between input and output layers for complex pattern learning.	Automatic feature extraction, high accuracy, scalability, and versatile applications characterise the system.	High computational cost, large data requirements, complexity with lack of interpretability, and susceptibility to overfitting are key challenges.	Computer vision, natural language processing (NLP), healthcare, and audio processing.
Decision Trees [61]	A supervised learning algorithm for classification and regression with intuitive decision paths.	The method offers interpretability, requires no data normalisation, and is robust to missing values.	Prone to overfitting and unstable with data variation.	Healthcare, finance, and marketing.
Instance-based classifiers [62]	Algorithms that predict using stored training examples.	Flexibility, adaptability, and simple implementation.	Computational complexity and sensitivity to noise.	Image recognition and text classification.
Support Vector Machine (SVM) [63]	A supervised algorithm for classification and regression tasks.	Effective for high-dimensional data and robust to overfitting.	The model faces challenges related to computational complexity and limited interpretability.	Bioinformatics and image classification.

**Table 4:** Summary of deep learning models for prostate cancer diagnosis.

Study	Modality	Model	Dataset Size	Key Metrics	Performance
[107]	MRI	Custom DL	400 patients	AUC	0.89 (cancer vs non-cancer), 0.78 (risk stratification)
[108]	DW-MRI	U-Net	112 patients	ICC, DSC	ICC: 0.80–0.83, DSC: 0.68–0.72
[109]	MRI + PSA	SNCSAE CAD	Not specified	Diagnostic Performance	Robust non-invasive detection
[110]	Histopathology	InceptionV3	96 biopsies	Accuracy, Sensitivity, Specificity	Acc: 92%, Sens: 90%, Spec: 93%
[111]	Histopathology	ResNet	85 biopsies	Accuracy	Coarse: 91.5%, Fine: 85.4%
[112]	MRI	ProGNet	905 patients	Dice Coefficient	Outperformed U-Net; 35s segmentation time
[114]	MRI	3D-Mask RCNN	Not specified	Accuracy	High accuracy
[115]	MRI	SEMRCNN	Not specified	Dice, Sensitivity, Specificity	Dice: 0.654, Sens: 0.695, Spec: 0.970
[116]	CT	DL + Multi-atlas	92 patients	Dice Coefficient	86.80%
[117]	MRI	Transfer Learning CNN	Not specified	Comparison with ML	DL superior to SVM, decision tree, Bayes
[118]	MRI	U-Net	165 patients	Dice Coefficient	0.22 (U-Net), 0.48–0.52 (manual)
[119]	MRI	U-Net	312 patients	Sensitivity, Specificity, Dice	Sens: 96%, Spec: 92%, Dice: 0.89

**Table 5:** Summary of prostate cancer and grand challenges in public datasets.

Dataset	Description	Modality	Sample Size	Key Application
PROSTATE158 [71]	High-resolution MRI scans for segmentation research	MRI (T2-weighted)	158 cases	Algorithm development for segmentation
Digital Pathology Dataset [116]	Whole Slide Images (H&E-stained); Tan Tock Seng Hospital	Histopathology	99 WSIs	Gland segmentation and classification
Peso Dataset [120]	WSIs with annotations; Radboud University Medical Centre	Histopathology	102 WSIs	Cancer detection and classification
TCGA Prostate Adenocarcinoma Dataset (TCGA-PRAD) [121]	Heterogeneous clinical data; multi-center collection	Mixed (Genomics, Imaging)	500 cases	Multi-omics analysis of prostate cancer
Gleason Challenge [122]	Tissue Microarrays (TMAs); Vancouver Prostate Center	Histopathology	244 training + 87 test TMAs	Gleason grading classification
PROSTATE MRI [NCI, USA] [123]	MRI guided biopsy and radical prostatectomy	MRI	Multiple cases	MRI-guided diagnostics and interventions
NCIGT-PROSTATE (NIH) [124]	MRI-guided therapy and biopsy procedures	MRI	Multiple cases	Image-guided therapy and biopsy research
PROMISE12 Challenge [125]	Segmentation challenge with ground-truth data	MRI (T2-weighted)	50 cases	Prostate gland segmentation benchmarking
QIN-PROSTATE-Repeatability [126]	Study on imaging biomarker reproducibility	MRI (T2W, DWI)	Multiple repeated scans	Biomarker validation and reproducibility
PANDA Challenge [127]	Largest publicly available prostate WSIs; Radboud & Karolinska	Histopathology	∼11,000 WSIs	AI development for Gleason grading

## Data Availability

No new data were created or analysed in this study. Data sharing is not applicable to this article.
